# Comparison of Imaging Modalities for Left Ventricular Noncompaction Morphology

**DOI:** 10.3390/jimaging11060185

**Published:** 2025-06-04

**Authors:** Márton Horváth, Dorottya Kiss, István Márkusz, Márton Tokodi, Anna Réka Kiss, Zsófia Gregor, Kinga Grebur, Kristóf Farkas-Sütő, Balázs Mester, Flóra Gyulánczi, Attila Kovács, Béla Merkely, Hajnalka Vágó, Andrea Szűcs

**Affiliations:** 1 Heart and Vascular Center, Semmelweis University, 1122 Budapest, Hungary; 2 Department of Biological Physics, Eötvös University, H-1117 Budapest, Hungary

**Keywords:** left ventricular noncompaction, cardiac imaging, echocardiography, cardiac MRI

## Abstract

Left ventricular noncompaction (LVNC) is characterized by excessive trabeculation, which may impair left ventricular function over time. While cardiac magnetic resonance imaging (CMR) is considered the gold standard for evaluating LV morphology, the optimal modality for follow-up remains uncertain. This study aimed to assess the correlation and agreement among two-dimensional transthoracic echocardiography (2D_TTE), three-dimensional transthoracic echocardiography (3D_TTE), and CMR by comparing volumetric and strain parameters in LVNC patients and healthy individuals. Thirty-eight LVNC subjects with preserved ejection fraction and thirty-four healthy controls underwent all three imaging modalities. Indexed end-diastolic, end-systolic, and stroke volumes, ejection fraction, and global longitudinal and circumferential strains were evaluated using Pearson correlation and Bland–Altman analysis. In the healthy group, volumetric parameters showed strong correlation and good agreement across modalities, particularly between 3D_TTE and CMR. In contrast, agreement in the LVNC group was moderate, with lower correlation and higher percentage errors, especially for strain parameters. Functional data exhibited weak or no correlation, regardless of group. These findings suggest that while echocardiography may be suitable for volumetric follow-up in LVNC after baseline CMR, deformation parameters are not interchangeable between modalities, likely due to trabecular interference. Further studies are warranted to validate modality-specific strain assessment in hypertrabeculated hearts.

## 1. Introduction

**Figure 1:**
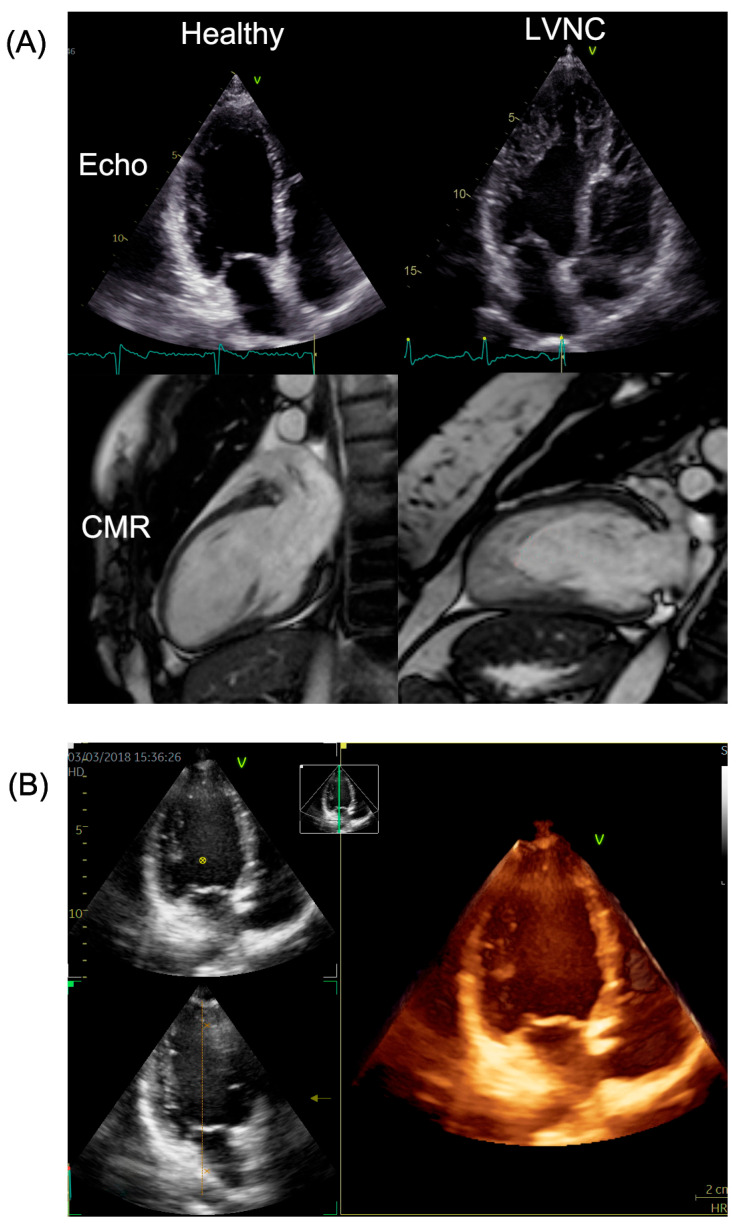
(**A**) 2D echo and CMR images of a healthy and an LVNC patient. (**B**) 3D echo image of a LVNC patient. LVNC: left ventricular non-compaction; CMR: cardiac magnetic resonance imaging. The orange line in figure (**B**) marks the 90° axis in the 3D echo image.

Excessive left ventricular trabeculation can be examined with multiple imaging modalities, of which cardiac magnetic resonance (CMR) is considered the gold standard for evaluating the huge amount of apical trabecular meshwork and establishing the diagnosis [1]. In certain cases, left ventricular noncompaction morphology (LVNC) develops to heart failure, emphasizing the significance of risk stratification and monitoring patients when deemed necessary. The deterioration of LVNC can be indicated by an increase in LV volumetric parameters and a decrease in LV functional parameters; however, the optimal modality for follow-up of this population remains unclear. Although CMR is known to be superior to echocardiography in measuring function and volume [2], the more readily available two-dimensional transthoracic echocardiography (2D_TTE) has a longer history in deformation analysis, which yields detailed information about subclinical functional changes of the LV. The optimal follow-up method should have a good cost–benefit ratio and be easily accessible and patient-friendly, such as modern three-dimensional transthoracic echocardiography (3D_TTE). This method has several advantages over two-dimensional LV strain measurements, as 3D strain values are derived from the entire LV myocardium, yielding more accurate estimates of global and regional LV function from a single beat image; thus, it is an easy-to-use option [3]. CMR and echo images of healthy and LVNC subjects are shown in Figure 1.

Several multimodal studies have reported that LVNC patients with preserved ejection fraction tend to exhibit increased volumetric parameters and mildly reduced strain values compared to healthy populations, even in the absence of overt dysfunction [4,5]. Despite the growing clinical use of echocardiography and CMR in the routine follow-up of LVNC patients, the direct comparison of these imaging modalities in this context has not yet been performed.

Given the methodological differences among modalities, such as tracking algorithms, spatial and temporal resolution, and strain calculation techniques, understanding the degree of agreement between them is of particular importance, especially in structurally altered hearts such as LVNC. CMR feature tracking, 2D speckle tracking, and 3D speckle tracking often yield systematically different strain values due to distinct computational approaches and acquisition geometries [6,7].

These considerations raise the question of which technique is more suitable for routine follow-up in LVNC. While several modality comparison studies have been conducted in other cardiomyopathies and general cardiac conditions, only limited data are available on hypertrabeculated populations. In particular, the level of agreement between different imaging techniques in both healthy and LVNC groups remains insufficiently explored. Therefore, our cross-sectional study aimed to compare volumetric and deformation parameters derived from CMR, 2D_TTE, and 3D_TTE in LVNC patients and healthy individuals, in order to evaluate intermodality agreement.

## 2. Materials and Methods

From the LVNC register of the Heart and Vascular Center of Semmelweis University, we selected 38 consecutive subjects with preserved left ventricular ejection fraction (EF > 50%) who underwent CMR, 2D_TTE, and 3D_TTE at the same checkup. The diagnosis was confirmed with cardiac magnetic resonance (MR) imaging. Two specific criteria had to be met for the diagnosis: the Petersen criteria, which required the ratio of non-compacted to compacted myocardial layer to exceed 2.3 at end-diastole, and the Jacquier criteria, which required the trabecular mass to be greater than 20% of the total myocardial mass at end-diastole.

Subjects with major comorbidities, congenital anomalies, coronary artery disease, or other cardiomyopathies were excluded. In addition to the hypertrabeculated LVNC population, we included 37 age- and sex-matched healthy volunteers without known cardiac or significant systemic disorders. Individuals engaging in more than 6 h of sports activity per week were also excluded.

All images were independently screened for feasibility prior to analysis. Echocardiographic recordings were excluded if endocardial borders could not be clearly delineated throughout the cardiac cycle due to suboptimal acoustic windows or overlapping lung tissue. In CMR, datasets affected by motion, arrhythmic or any other artifacts, poor temporal resolution, or usage of contrast agent before the acquisition of the short axis images were also excluded. No significant differences in image quality or feasibility were observed between healthy and LVNC participants among the included cases. The baseline characteristics of the study population are shown in the Supplementary Materials.

All the CMR and echocardiographic image acquisitions were performed in accordance with the guidelines of the European Society of Cardiology. CMR scans were carried out using either a 1.5-T Philips Achieva system (Philips Medical System, Eindhoven, The Netherlands) or a Siemens Magnetom Aera MRI system (Siemens Healthineers AG, Erlangen, Germany), both equipped with a 5-channel cardiac coil. Electrocardiogram (ECG)-gated, balanced steady-state free precession (bSSFP) sequences were acquired in 2-, 3-, and 4-chamber long-axis views, supplemented by breath-hold short-axis stacks extending from the base to the apex. Acquisition parameters for the Philips and Siemens scanners were as follows: repetition times of 2.7 ms and 2.5 ms, echo times of 1.3 ms and 1.15 ms, flip angles of 60° and 58°, spatial resolution of 1.5 × 1.5 mm^2^, and a temporal resolution of 25 frames per cardiac cycle for both systems. Slice thickness was maintained at 8 mm without interslice gaps, and the field of view was adapted to approximately 350 mm according to patient size. Volumetric and functional analyses were conducted using the semiautomatic-contouring QMass module (Medis Suite, version 3.0, Medis Medical Imaging Systems, Leiden, The Netherlands) with manual adjustment. Endocardial contours were traced from base to apex on cine short-axis images, following the compact–noncompact interface to include trabeculae within the LV cavity. Feature tracking-based strain assessment was performed using the QStrain module on 2-, 3-, and 4-chamber long-axis images. Global circumferential strain (GCS) values were computed based on contours previously defined in short-axis views, while global longitudinal strain (GLS) was calculated from automated contours on long-axis views.

2D_TTE and 3D_TTE examinations were performed using a GE Vivid E95 ultrasound system with a 4Vc-D phased-array transducer (GE Vingmed Ultrasound, Horten, Norway). ECG-gated, LV-focused apical long-axis, four-chamber, and two-chamber loops were acquired at frame rates exceeding 50 frames per second. For the 2D analysis, the TOMTEC Cardiac Performance Analysis software (TOMTEC Imaging Systems GmbH, Unterschleissheim, Germany) was used. This semi-automated software generated endocardial contours, which were manually refined as needed to ensure accurate border tracking.

For 3D_TTE, full-volume datasets were acquired using multibeat reconstruction from four cardiac cycles in the apical four-chamber view. Offline analysis was performed using the 4D LV Analysis software (TOMTEC Imaging Systems GmbH, Munich, Germany), selecting the optimal cardiac cycle. The algorithm generated 3D endocardial contours, which were manually adjusted across short- and long-axis planes throughout the cardiac cycle if it was necessary. In both echocardiographic modalities, contours were aligned along the compact-noncompact boundary to incorporate trabeculations into the LV cavity. Speckle tracking was applied to derive GLS and GCS values.

Based on CMR and echocardiographic data, end-diastolic volume (EDV), end-systolic volume (ESV), and stroke volume (SV) were indexed to body surface area using the Du Bois formula. Ejection fraction (EF), GLS, and GCS were also evaluated. CMR-based volumetric and strain analyses were independently performed by two observers (A.Sz., with 9 years of experience, and A.R.K., with 5 years of experience). The 2D and 3D echocardiographic datasets were analyzed by two observers (A.Sz., with 5 years of experience, and M.H., with 3 years of routine experience).

The Shapiro–Wilk test was used to assess the normality of data distributions. An unpaired two-sided Student’s *t* test, in normal distribution, or a Mann–Whitney U test, in nonnormal distribution, was performed to compare the continuous variables of the study groups.

Interobserver agreement was tested and presented using the interclass correlation coefficient (ICC) and 95% confidence intervals. A *p* value of <0.05 was used as the criterion for statistical significance.

Pearson correlation was used to examine the correlation between modalities. When evaluating correlations, these were rated as weak when below 0.3, moderately good when between 0.3 and 0.6, and excellent when above 0.6.

Bland–Altman analysis was used to determine intermodality agreement [8]. For further quantification of their relative differences, we used percentage error (PE), which is the ratio between the magnitude of the measurement and the measurement error (derived by dividing the limits of agreement by the mean value of the measurements) [9]. The magnitude of acceptable PE depends on the measured variable [2]. For the assessment of PE, a cutoff value of 0.3 was used, which is a commonly applied threshold for acceptable agreement between two methods in clinical usage [10]. The results below this cutoff value were considered a good match.

We used SPSS v.28.0. (IBM, New York, NY, USA) and Python v.3.11. software to compile the statistics.

## 3. Results

The interobserver agreement on the Medis Qmass module, the CMR feature tracking, and both 2D and 3D TOMTEC software was analyzed in ten randomly selected LVNC participants and ten healthy subjects. The exact results of the interobserver variability test can be found in the Supplementary Materials.

Considering the volumes measured by 2D_TTE, 3D_TTE, and CMR, we found that the LVNC group had higher volumetric parameters than the healthy group with all modalities (Table 1).

Regarding the volumetric parameters, we compared the three modalities in both the patient and healthy groups by calculating pairwise correlations and using a Bland–Altman analysis. We found an excellent correlation and good agreement among the three techniques in the healthy subjects. The results are summarized in Table 2. In the LVNC group, we were also able to confirm moderate to high correlations for EDV(i) and SV(i), whereas for the values of ESV(i), only weaker relationships could be determined between echo and CMR modalities, which could be caused by the vague visibility of the trabeculae in end-systole. The percentage errors, according to the Bland–Altman analysis, also showed similar patterns. Notably, 3D_TTE and CMR measurements agreed well for volumetric data with correlation coefficient near 0.9, and most of the percentage errors were well below our acceptance limit of 0.3.

EF and strain values had fewer and weaker correlations and higher percentage errors among modality pairwise comparisons in both groups. However, similar to the results for volumetric data, agreement between the modalities was even less frequent in the LVNC group. The summary of the Bland–Altman analysis and correlations in both the healthy and LVNC groups are shown in Table 3.

**Figure 2:**
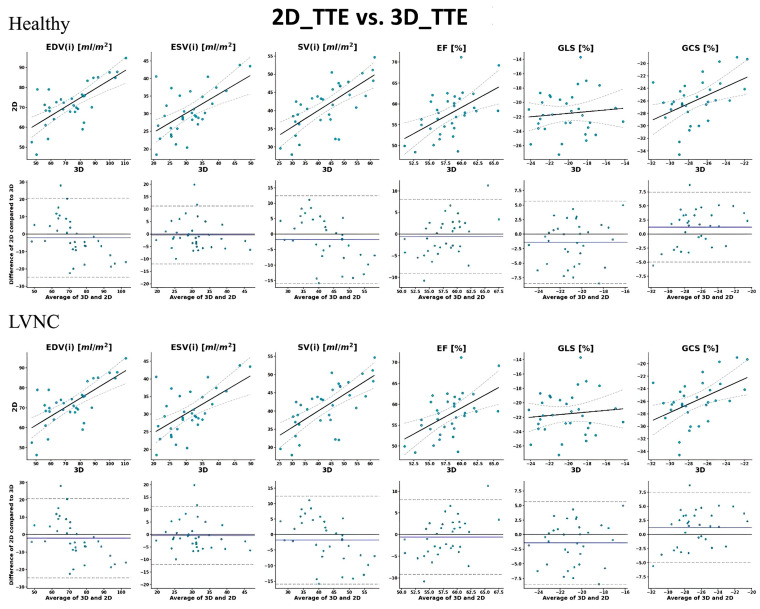
Correlation and Bland–Altman plots between 2D echocardiography and 3D echocardiography imaging-derived measurements. In the upper row, solid lines represent the linear regression fit, while dashed lines indicate the 95% confidence intervals of the regression. In the lower row (Bland–Altman plots), the solid line shows the mean difference between modalities, and dashed lines represent the 95% limits of agreement (±1.96 SD). Each dot corresponds to an individual measurement. EDV: end-diastolic volume; ESV: end-systolic volume; SV: stroke volume; EF: ejection fraction; GLS: global longitudinal strain; GCS: global circumferential strain; and (i): indexed to body surface area.

**Figure 3:**
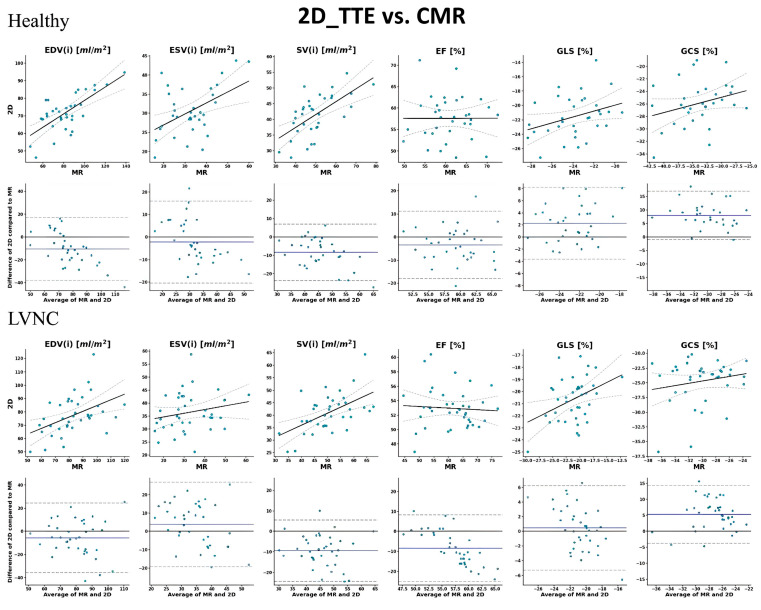
Correlation and Bland–Altman plots between 2D echocardiography and CMR imaging-derived measurements. In the upper row, solid lines represent the linear regression fit, while dashed lines indicate the 95% confidence intervals of the regression. In the lower row (Bland–Altman plots), the solid line shows the mean difference between modalities, and dashed lines represent the 95% limits of agreement (±1.96 SD). Each dot corresponds to an individual measurement. EDV: end-diastolic volume; ESV: end-systolic volume; SV: stroke volume; EF: ejection fraction; GLS: global longitudinal strain; GCS: global circumferential strain; and (i): indexed to body surface area.

**Figure 4:**
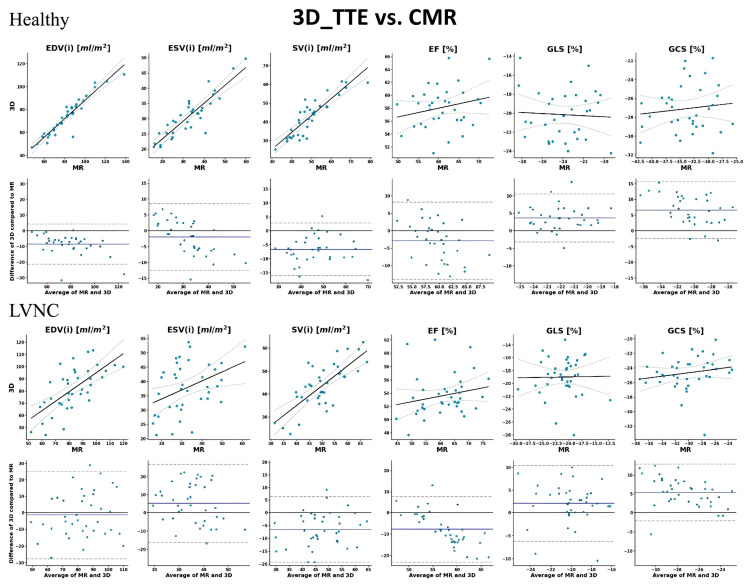
Correlation and Bland–Altman plots between 3D echocardiography and CMR imaging-derived measurements. In the upper row, solid lines represent the linear regression fit, while dashed lines indicate the 95% confidence intervals of the regression. In the lower row (Bland–Altman plots), the solid line shows the mean difference between modalities, and dashed lines represent the 95% limits of agreement (±1.96 SD). Each dot corresponds to an individual measurement. EDV: end-diastolic volume; ESV: end-systolic volume; SV: stroke volume; EF: ejection fraction; GLS: global longitudinal strain; GCS: global circumferential strain; and (i): indexed to body surface area.

Importantly, although we found a similar number of significant (*p* < 0.05) correlations in both groups, the strengths and confidences were generally lower for the LVNC group. In line with these findings, there was a greater agreement for all three modality pairs in the healthy group (16 out of 18 values; 89%) than in the patient population (8 out of 18 values; 38%). Moreover, we need to mention that volumetric data in both groups were generally underestimated by 2D_TTE and 3D_TTE compared to CMR and by 2D_TTE compared to 3D_TTE. The correlation and Bland–Altman plots between 2D echocardiography, 3D echocardiography, and CMR imaging-derived measurements are shown in Figure 2, Figure 3 and Figure 4.

## 4. Discussion

This study was designed as a cross-sectional analysis to explore intermodality agreement under standardized conditions, as its findings may provide a foundation for planning the longitudinal follow-up of LVNC patients.

Choosing an appropriate imaging modality for the follow-up of LVNC, which requires the measurement of volumetric and functional parameters, is still an open question and requires many aspects to be considered. Therefore, it is important to investigate how the results obtained with different techniques differ from each other. The comparison of modalities revealed significant differences in the measured volumetric and functional parameters between the two groups, which is consistent with the literature [11]. Importantly, the fundamental methodological differences between CMR feature tracking and both 2D and 3D speckle tracking echocardiography limit the direct comparability of volumes and strain values, particularly in pathological conditions such as LVNC.

To our knowledge, the only intermodal comparative study in the literature on noncompaction found CMR to be clearly superior to 2D_TTE in determining the extent of trabeculae. However, this study did not include volumes or cardiac function and was only performed on one group of patients [1].

In our study, we conducted a comparative analysis of volumetric and functional parameters using diverse modalities both in LVNC and healthy groups, assessing the correlation and agreement among these techniques.

In the healthy group, volumetric data showed strong correlations and good agreement between all modalities with almost one-to-one interchangeability of 3D_TTE and CMR. Although we found significant correlations in the LVNC group, the level of agreement was suboptimal between the techniques.

A number of studies in the literature to date compare imaging techniques, mainly in a single patient group. A study compared 2D_TTE and 3D_TTE in a population of patients after cardiac resynchronization therapy and found a very good correlation between the two modalities in terms of volumetric data, which is in line with our results in both the healthy and LVNC groups [12]. However, in contrast to the study by Hotta, we did not find significant correlations of EF values between the examined modalities (except when comparing 2D_TTE with 3D_TTE in the healthy group), even though the percentage errors of the Bland–Altman analysis were still in the acceptable range. The latter confirms that differences between the modalities are relatively small, and the absence of correlations can be explained by the narrow range of EF values. This, however, might mean that modalities are not interchangeable for tracking small changes in EF, as in our groups.

Another study on heart failure also found a moderate-to-strong correlation between CMR and 2D_TTE volumetric results, but interestingly, this correlation was highly dependent on the degree of left ventricular dysfunction [13]. Several investigations of ischemic heart disease have concluded that CMR and echocardiography volumetric results correlate moderately well but 2D_TTE in general yields lower values than CMR, as in our investigation [12,14]. A study performed in a hypertrophic cardiomyopathy (HCM) population concluded that volumetric results of 2D_TTE and 3D_TTE had good agreement; nonetheless, agreement was found to be only moderate for EF and slightly underestimated by 2D_TTE. Moreover, this study found a similarly good agreement between volumetric data in the 3D_TTE-CMR comparison [15]. These agreements are in line with our results mostly in the healthy population, with underestimation when using echocardiography. A study comparing 3D_TTE and CMR in several groups of patients (aortic valve disease, mitral regurgitation, previous myocardial infarction, and healthy volunteers) concluded that 3D_TTE was comparable to CMR in terms of volumetric data in the presence and absence of wall motion abnormalities with underestimation of 3D echo [16]. These results are in line with those of our healthy population; however, volumes in the LVNC group did not agree although the correlation was good between the tested modalities. This systematic underestimation of volumes by echocardiographic techniques compared to CMR has been reported previously in various patient populations [12,14]. However, in the presence of excessive trabeculation, as in LVNC, this discrepancy may be more pronounced due to the difficulty in delineating endocardial borders. Therefore, while echocardiography, particularly 3D_TTE, may be suitable for serial volume assessment, it is advisable to use the same modality consistently in hypertrabeculated patients to avoid misinterpretation.

The reason for this discrepancy may be that they did not observe pathology with hypertrabeculated conditions, and an echo contrast agent was used to enhance visualization, which can increase the concordance of the results. To the best of our knowledge, this is the only study that compared multiple modalities in different groups, similar to our study, but strain parameters were not considered.

For the strain analysis, in contrast to the healthy population, the LVNC group had only poor agreement; furthermore, only a sporadic relationship was found between feature and speckle tracking strain parameters dominantly in the LVNC group. The mainly poor correlation of functional parameters of both groups could be due to the small variance in data, which was also mentioned above in connection with EF. Notably, GLS and GCS derived from the 3D surface are methodologically completely different from a feature or 2D speckle tracking echocardiography, which might also cause alterations in the agreement.

Pryds et al. investigated different groups of patients, e.g., patients with compensated heart failure, acute perimyocarditis, aortic stenosis, heart transplantation, and healthy volunteers, and found that global strains measured by CMR feature tracking correlated with the results measured by speckle tracking echocardiography, but this correlation was not consistent across subgroups. Therefore, they suggest that feature tracking and speckle tracking techniques should not be used interchangeably in the evaluation of myocardial strains in these pathologies [6]. Erley et al. investigated intermodal agreement in ischemic, nonischemic, and healthy groups and revealed good agreement between groups, as in our healthy population [17]. Moreover, the same investigator observed the right ventricles of various patient populations, which can be normally as trabeculated as the left ventricle in the case of LVNC and found less agreement between modalities of strain values. These findings support the theory that different modalities are not interchangeable in deformation analysis [7].

In summary, the correlation and agreement of volumes were excellent in the healthy population and acceptable in the case of LVNC; however, the weak relationship and agreement of our LVNC group suggest that hypertrabecularization may cause difficulties when comparing strains measured with different modalities.

Clinically, percentage errors exceeding the 30% threshold may limit the modality’s utility for detecting subtle functional changes over time. In LVNC, where early deterioration can precede overt dysfunction, high PE values in strain suggest that modalities should not be used interchangeably. Modality-specific reference values and follow-up strategies may therefore be necessary for reliable monitoring.

Thus, on the basis of our results, ultrasound follow-up is possible after diagnostic CMR examination, whereas the measurement of strains that are representative of subclinical changes is not interchangeable between modalities in excessive trabeculation. This observation has important implications for routine clinical follow-up in LVNC patients, particularly when echocardiographic strain imaging is used to detect early functional changes. Given the limited agreement in strain parameters, modality-specific reference values and longitudinal assessments are recommended to avoid misinterpretation.

In the literature, only limited data are available on this topic. Therefore, more sophisticated imaging techniques and larger, multimodal studies are needed to validate the comparability of strain parameters across modalities in specific cardiac pathologies, particularly in hypertrabeculated populations.

## 5. Conclusions

In the present study, we investigated the correlation and agreement of 2D_TTE and 3D_TTE and CMR modalities in healthy and LVNC groups to determine the optimal technique for LVNC patient follow-up.

In the healthy group, volumetric measurements agreed excellently between different modalities in terms of correlations and percentage errors calculated from the Bland–Altman analysis. For volumetric data, 3D_TTE and CMR modalities matched well, having an almost one-to-one correspondence. In terms of functional parameters, relative differences were found to be acceptable; however, significant strong correlations could not be established.

For the LVNC group, we were able to show significant correlations between modalities when measuring volumetric data, but their relative differences proved to be higher than our acceptance criteria, which indicates that methods are not replaceable. Furthermore, for functional parameters, we did not observe sufficient coherence in our results, which would suggest interchangeability of modalities in global strain measurements.

Our conclusions indicate that hypertrabeculation might cause difficulties in the comparison of recordings. Based on our findings, 3D transthoracic echocardiography may be acceptable for volumetric monitoring following a diagnostic CMR scan. However, global strain measurements appear to be modality-dependent and should only be used for longitudinal follow-up when performed consistently using the same imaging technique.

Limitations: one major limitation of our research is the relatively small number of cases. LVNC is a rather rare disease. Our registry is constantly expanding; however, a prospective, multimodal analysis of this (geographically also dispersed) population is not easily accessible. A notable limitation is that although 2D-STE and MR-FT similarly compute strains, 3D-STE derives data from a 3D surface, which is a methodologically completely different method. Thus, numerical comparison of parameters can be difficult. As mentioned in the article, we only worked with populations with preserved left ventricular function for good comparability, resulting in minimal standard deviations for ejection fraction, GLS and GCS; thus, the accuracy of the correlation in these cases is questionable. Our findings apply specifically to LVNC patients with preserved left ventricular function. The applicability of these results to patients with reduced ejection fraction remains uncertain and should be addressed in future studies. The present study did not utilize ultrasound contrast agents; however, their use could have improved the visualization. Based on these cross-sectional pilot studies, our plan is the longitudinal investigation of this patient population including clinical outcome data in a multicentric design.

## Figures and Tables

**Table 1:** Baseline characteristics of examined groups.

	Healthy			LVNC			*p* Value Healthy vs. LVNC		
Number of Patients (Male)	38 (25)			34 (19)					
Age (Years)	36 ± 13			31 ± 14					
	2D_TTE	3D_TTE	CMR	2D_TTE	3D_TTE	CMR	2D_TTE	3D_TTE	CMR
EDV(i) (mL/m^2^)	72.1 ±10.4	74.1 ± 16.3	82.5 ± 19.2	77.4 ± 14.2	81.8 ± 17.7	82.9 ± 15.3	0.005 *	0.05 *	0.1
ESV(i) (mL/m^2^)	30.6 ± 6.3	30.9 ± 6.9	32.8 ± 10.5	36.5 ± 7.3	37.9 ± 8.7	32.6 ± 8.4	<0.01 *	<0.01 *	0.3
SV(i) (mL/m^2^)	41.5 ± 6.5	43.1 ± 10.1	49.7 ± 10	40.9 ± 7.4	43.8 ± 9.7	50.2 ± 8.5	0.1	0.1	0.09
EF (%)	57.5 ± 5	58.1 ± 3	60.9 ± 5	52.9 ± 3	53.6 ± 3	61.3 ± 8	<0.01 *	<0.01 *	0.8
GLS (%)	−21.5 ± 2.9	−20.2 ± 2.5	−23.9 ± 2.3	−20.6 ± 1.8	−18.9 ± 2.9	−21.1 ± 3.1	0.1	0.07	<0.01 *
GCS (%)	−25.9 ± 3.5	−27.1 ± 2.5	−33.8 ± 4.1	−24.7 ± 3.7	−24.6 ± 2.3	−30.1 ± 3.5	0.2	<0.01 *	<0.01 *

LVNC: left ventricular non-compaction; TTE: transthoracic echocardiography; CMR: cardiac magnetic resonance imaging; EDV: end-diastolic volume; ESV: end-systolic volume; SV: stroke volume; EF: ejection fraction; GLS: global longitudinal strain; GCS: global circumferential strain; (i): indexed to body surface area; and *: *p* < 0.05.

**Table 2:** Results of the correlation and Bland–Altman analysis of the modality pairs in LVNC patients and healthy volunteers.

(A) Healthy						(B) LVNC					
2D_TTE vs. 3D_TTE						2D_TTE vs. 3D_TTE					
	Correlation		Bland–Altman				Correlation		Bland–Altman		
	r	*p*<	Bias	LOA	PE (%)		r	*p*<	Bias	LOA	PE (%)
EDV(i)	0.71	0.01	−1.978 *	22.7	29.65	EDV(i)	0.73	0.01	−4.341 *	23.7	28.95
ESV(i)	0.59	0.01	−0.286 *	11.6	25.65	ESV(i)	0.7	0.01	−1.426	12.4	32.65
SV(i)	0.69	0.01	−1.692*	14.2	27.9	SV(i)	0.66	0.01	−2.915 *	14.5	33
EF	0.53	0.01	−0.005	0.9	14.78	EF	0.05	0.79	−0.007	0.08	14.66
GLS	0.11	0.55	−1.403 *	7.1	35.19	GLS	0.17	0.29	−1.627 *	45828	32.72
GCS	0.48	0.01	1.282 *	6.2	22.78	GCS	0.12	0.47	−0.068	8	32.58
2D_TTE vs. CMR						2D_TTE vs. CMR					
	Correlation		Bland–Altman				Correlation		Bland–Altman		
	r	*p*<	Bias	LOA	PE (%)		r	*p*<	Bias	LOA	PE (%)
EDV(i)	0.71	0.01	−10.42 *	27.4	29.2	EDV(i)	0.46	0.01	−5.476 *	30.1	36.30
ESV(i)	0.48	0.01	−2.176	18.2	55.4	ESV(i)	0.21	0.19	3.834	23	70.50
SV(i)	0.63	0.01	−8.246 *	15.4	28.9	SV(i)	0.55	0.01	−9.309 *	14.9	29.70
EF	0.01	0.99	−0.033 *	0.14	24	EF	−0.06	0.7	−0.084 *	0.16	27.20
GLS	0.34	0.05	2.281 *	5.9	24.90	GLS	0.39	0.02	0.491	5.8	27.30
GCS	0.29	0.01	8.074 *	9	26.4	GCS	0.19	0.26	5.37	9.1	30.10
3D_TTE vs. CMR						3D_TTE vs. CMR					
	Corrrelation		Bland–Altman				Correlation		Bland–Altman		
	r	*p*<	Bias	LOA	PE (%)		r	*p*<	Bias	LOA	PE (%)
EDV(i)	0.94	0.01	−8.444 *	12.9	15.62	EDV(i)	0.67	0.01	−1.135	26.5	31.93
ESV(i)	0.89	0.01	−1.89 *	10.5	29.98	ESV(i)	0.39	0.02	5.26 *	21.5	65.83
SV(i)	0.88	0.01	−6.555 *	9.4	18.97	SV(i)	0.75	0.01	−6.394 *	12.8	25.45
EF	0.23	0.19	−0.028 *	0.1	18.14	EF	0.23	0.19	−0.077 *	0.2	25.02
GLS	−0.05	0.76	3.709 *	6.9	28.71	GLS	0.014	0.93	2.117 *	8.4	39.63
GCS	0.12	0.51	6.683 *	9	26.68	GCS	0.19	0.26	5.438 *	7.5	24.94

LVNC: left ventricular non-compaction; TTE: transthoracic echocardiography; CMR: cardiac magnetic resonance imaging; EDV: end-diastolic volume; ESV: end-systolic volume; SV: stroke volume; EF: ejection fraction; GLS: global longitudinal strain; GCS: global circumferential strain; (i): indexed to body surface area; LOA: limits of agreement; PE: percentage error; and *: *p* < 0.05.

**Table 3:** Summary of the agreement and correlation data. A+: acceptable agreement; A−: unacceptable agreement; C+: acceptable correlation; C−: unacceptable correlation; EDV: end-diastolic volume; ESV: end-systolic volume; SV: stroke volume; EF: ejection fraction; GLS: global longitudinal strain; GCS: global circumferential strain; and (i): indexed to body surface area.

	Healthy			LVNC		
	2D_TTE–3D_TTE	2D_TTE–CMR	3D_TTE–CMR	2D_TTE–3D_TTE	2D_TTE–CMR	3D_TTE–CMR
A+, C+	EDV(i), ESV(i), SV(i), EF, GCS	EDV(i), SV(i), GLS	EDV(i), ESV(i), SV(i)	EDV(i)	SV(i), GLS	SV(i)
A−, C+		ESV(i)		ESV(i), SV(i)	EDV(i)	EDV(i), ESV(i)
A+, C−		EF, GCS	EF, GLS(i), GCS(i)	EF	EF,	EF, GCS
A−, C−	GLS			GLS, GCS	ESV(i), GCS	GLS

## Data Availability

The data presented in this study are available on reasonable request from the corresponding author. The data are not publicly available due to privacy or ethical restrictions.

## Supplementary Materials

The following supporting information can be downloaded at: https://www.mdpi.com/article/10.3390/jimaging11060185/s1.

## Abbreviations

2D_TTE	Two-dimensional transthoracic echocardiography
3D_TTE	Three-dimensional transthoracic echocardiography
A+	Acceptable agreement
A−	Unacceptable agreement
bSSFP	Balanced steady-state free precession
C+	Acceptable correlation
C−	Unacceptable correlation
CMR	Cardiac magnetic resonance imaging
ECG	Electrocardiogram
EDV	End-diastolic volume
EDV(i)	End-diastolic volume indexed to body surface area
EF	Ejection fraction
ESV	End-systolic volume
ESV(i)	End-systolic volume indexed to body surface area
GCS	Global circumferential strain
GLS	Global longitudinal strain
H	Healthy
HCM	Hypertrophic cardiomyopathy
ICC	Intraclass correlation coefficient
I	Indexed to body surface area
LOA	Limits of agreement
LV	Left ventricle/left ventricular
LVNC	Left ventricular noncompaction
MRI	Magnetic resonance imaging
MR-FT	Magnetic resonance feature tracking
PE	Percentage error
SV	Stroke volume
SV(i)	Stroke volume indexed to body surface area
STE	Speckle tracking echocardiography
TTE	Transthoracic echocardiography
