# Supplementary material for: Comparison of Imaging Modalities for Left Ventricular Noncompaction Morphology
Source: J Imaging. 2025 Jun 4;11(6):185. doi: 10.3390/jimaging11060185 (PMC12194762; doi:10.3390/jimaging11060185)
Supplement: Supplementary file 1 [file jimaging-11-00185-s001.zip › jimaging-3641477-supplementary.pdf]

Supplementary material

	3D_TTE	2D_TTE	CMR
**EDV(i)**	0.96 (0.80–0.99)	0.67 (0.56–0.73)	0.98 (0.95–0.99)
**ESV(i)**	0.95 (0.76–0 99)	0.98 (0.81–0. 99)	0.94 (0.84–0 98)
**SV(i)**	0.89 (0.52–0.98)	0.90 (0.53–0.98)	0.90 (0.75–0.96)
**EF**	0.65 (0.58–0.92)	0.89 (0.51–0.98)	0.76 (0.40–0.91)
**GLS**	0.53 (0.48–0.89)	0.93 (0.67–0.98)	0.96 (0.89–0.98)
**GCS**	0.58 (0.49–0.79)	0.89 (0.52–0.98)	0.96 (0.89–0.98)

The results of the interobserver agreement

Interobserver agreement was rated as weak below 0.4, moderately good between 0.4 and 0.75, and excellent above 075.

TTE: Transthoracic echocardiography; CMR: Cardiac Magnetic Resonance Imaging; EDV: End-diastolic volume; ESV: End-systolic volume; SV: Stroke volume; EF: Ejection fraction; GLS: Global longitudinal strain; GCS: Global circumferential strain; (i): indexed to body surface area
